# Genome comparison using Gene Ontology (GO) with statistical testing

**DOI:** 10.1186/1471-2105-7-374

**Published:** 2006-08-11

**Authors:** Zhaotao Cai, Xizeng Mao, Songgang Li, Liping Wei

**Affiliations:** 1 Center for Bioinformatics, National Laboratory of Protein Engineering and Plant Genetic Engineering, College of Life Sciences, Peking University, Beijing 100871, P.R. China

## Abstract

**Background:**

Automated comparison of complete sets of genes encoded in two genomes can provide insight on the genetic basis of differences in biological traits between species. Gene ontology (GO) is used as a common vocabulary to annotate genes for comparison. Current approaches calculate the fold of unweighted or weighted differences between two species at the high-level GO functional categories. However, to ensure the reliability of the differences detected, it is important to evaluate their statistical significance. It is also useful to search for differences at all levels of GO.

**Results:**

We propose a statistical approach to find reliable differences between the complete sets of genes encoded in two genomes at all levels of GO. The genes are first assigned GO terms from BLAST searches against genes with known GO assignments, and for each GO term the abundance of genes in the two genomes is compared using a chi-squared test followed by false discovery rate (FDR) correction. We applied this method to find statistically significant differences between two cyanobacteria, *Synechocystis *sp. PCC6803 and *Anabaena *sp. PCC7120. We then studied how the set of identified differences vary when different BLAST cutoffs are used. We also studied how the results vary when only subsets of the genes were used in the comparison of human *vs*. mouse and that of *Saccharomyces cerevisiae *vs. *Schizosaccharomyces pombe*.

**Conclusion:**

There is a surprising lack of statistical approaches for comparing complete genomes at all levels of GO. With the rapid increase of the number of sequenced genomes, we hope that the approach we proposed and tested can make valuable contribution to comparative genomics.

## Background

Comparison of two completely sequenced genomes sheds lights on the genetic basis of differences in biological traits between species. Of particular interest is the comparison of complete sets of genes and gene products encoded in two genomes. Manual comparison is important but time-consuming and labor-intensive at the whole-genome scale and thus must be aided by automated approaches.

Unambiguous automated comparison requires that both genomes be annotated with the same structured, controlled vocabulary. Currently, the most common choice for such a vocabulary is gene ontology (GO) [1]. The November 15, 2005 version of GO contained 19,025 terms in three hierarchical structures—as Directed Acyclic Graphs (DAGs)—termed Biological Processes, Cellular Components, and Molecular Functions. Every branch in the graph represents a biological concept progressing from general to specialized with increasing graph depth. The depth of the branches in the graphs varies, with levels ranging from 2 to 15.

The GO web site currently lists 31 genomes that have been annotated with GO [2]. The annotations that are of the highest quality and updated most frequently are usually carried out by researchers who sequence and study a particular species; these annotations are primarily stored in species-specific databases such as SGD [3] for *Saccharomyces cerevisiae*, FlyBase [4] for *Drosophila melanogaster*, WormBase [5] for *Caenorhabditis elegans*, MGI [6] for *Mus musculus*, and TAIR [7] for *Arabidopsis thaliana*. Since these species-specific databases are located in different sites on the web, there is need for integrated, searchable databases that contain annotations for multiple species. The GO Consortium has developed such a resource, called AMIGO [8], that allows users to search and browse GO annotations integrated from many species-specific databases. Additionally, the European Bioinformatics Institute (EBI) has developed the Gene Ontology Annotation (GOA) database [9] that provides GO annotations for non-redundant proteins from many species in UniProt [10,11]. We compare these two resources in the Methods section. In addition to sequences annotated with GO, 15,754 functional domains in the InterPro domain database [12] have been linked to 2,627 GO terms [13].

Using the above-mentioned resources, there are two main types of methods developed to automatically annotate new gene products with GO terms: sequence similarity-based methods such as GOFigure [14], Goblet [15], OntoBlast [16], GOtcha [17], and Blast2GO [18], and sequence domain-based methods such as InterProScan [19] and GOTrees [20]. For genome-scale GO annotations the similarity-based, in particular BLAST-based methods have been the preferred choice [17,21-24]. BLAST is significantly faster than InterProScan and can annotate many more GO terms than InterProScan can. A recent evaluation showed that assigning GO terms of the top BLAST hit gave satisfactory results when compared with several more complex methods [25]. Thus we chose the BLAST approach in our work.

After the sets of genes encoded in the two genomes are annotated with GO, they can then be compared. The goal is to find functional categories that differ between the two genomes, which may explain differences in biological traits or suggest interesting families for further detailed investigation. The most common practice is to use tools such as GOslim [26,27] to tally the number of genes that fall within each functional category at the first level under Biological Processes, Cellular Components, and Molecular Functions, and then to compare between the two genomes. Because the two genomes usually differ in size, the absolute numbers of genes in each functional category need to be weighted before they are compared; they are often divided by the total number of genes in the respective genomes [28-30]. The results of the unweighted and weighted comparisons are usually presented as bar charts or fold changes.

The unweighted and weighted GO-based genome comparisons, although useful, have two drawbacks. First, focusing only on the high-level functional categories may miss differences that are detectable only at more refined levels. Second, bar charts or fold changes alone are not sufficient to separate true functional differences from those occurring by chance; thus, statistical testing of significance is necessary. Lessons can be learned from another, more extensively researched application of GO—the detection of significantly enriched GO categories in a set of co-expressed or differentially expressed genes in microarray experiments. Several tools have been developed to search complete GO trees (rather than just the high levels) and apply statistical testing of significance (e.g., Onto-Express [31]; FatiGO [32]; for an evaluation of these tools, see ref. [33]).

Contrary to the situation in microarray analysis, there is a surprising lack of statistical approaches for GO-based comparison of two genomes. Here we propose such a statistical approach to find reliable differences between the complete sets of genes encoded in two genomes at all levels of GO. For each GO term the abundance of genes in the two genomes is compared using chi-squared test followed by false discovery rate (FDR) correction. Furthermore, to analyze the reliability of the differences detected, we studied two important issues. First, when new sequences are assigned GO terms by similarity (as determined by BLAST) to other sequences having known GO assignments, the choice of BLAST cutoff may affect the results. We therefore analyzed the effects of employing a wide range of BLAST cutoffs. Second, we studied how the results vary when only subsets of the genes were used. To our knowledge, our work is the first to address all the aforementioned issues.

We used this statistical approach to compare two cyanobacterial genomes, *Synechocystis *sp. PCC6803 and *Anabaena *sp. PCC7120. Cyanobacteria (also called blue-green bacteria, blue-green algae, cyanophyceae, or cyanophytes) are important model organisms for the study of photosynthesis, nitrogen fixation, evolution of plant plastids, and survival in diverse environments [34-41]. Two of the most widely studied cyanobateria species are *Synechocystis *sp. PCC6803 and *Anabaena *sp. PCC7120. PCC6803 is a fresh water unicellular cyanobacterium incapable of nitrogen fixation [42]; PCC7120 is a filamentous, heterocyst-forming cyanobacterium that has long been used to study the genetics and physiology of cellular differentiation, pattern formation, and nitrogen fixation [43]. These interesting biological differences as well as the appropriate evolutionary distance between PCC6803 and PCC7120 make them a popular pair of species to compare and contrast[34,44-50]. We compared PCC6803 and PCC7120 genomes using our statistical method and evaluated the detected statistically significant differences against known biological differences. To analyze how results change when only subsets of the genes are used, a larger set of statistically significant differences is desirable and we used the comparison of human *vs*. mouse and that of *Saccharomyces cerevisiae vs*. *Schizosaccharomyces pombe *genomes.

## Results

### Whole-genome GO annotation

To annotate a new sequence, we used BLAST to compare it against a database of sequences with known GO annotations. Such a database should contain as many annotated sequences as possible from as many species as possible. AMIGO and GOA are two primary choices for such a database. We compared AMIGO and GOA, as shown in Table 1. Both databases have unique merit. AMIGO has been integrated to a greater extent with other databases and provides a better browsing function on the web, whereas GOA contains more sequences. For our purpose, it was attractive to have a larger collection of sequences for comparisons using BLAST, and thus we chose the GOA database. We set the default BLAST cutoff E-value to be 1E-20. With this method, a gene is assigned the GO terms of its top BLAST hit in GOA; it is also linked to all parent GO terms by propagating the DAG structures. Finally, the number of genes assigned to each GO term is tallied, representing the abundance of genes in each GO function within the genome.

We were able to annotate 2,224 genes in the PCC6803 genome to 1,933 GO terms, and 3,348 genes in the PCC7120 genome to 1,947 GO terms.

### Testing the statistical significance of detected differences between genomes

For each GO category, we used the chi-squared test to determine whether the numbers of genes from the two genomes were statistically significantly different [51]. Since the total number of GO categories is large, a large number of tests is required. We adopted the widely used FDR correction (*q*-value cutoff = 0.01) to control the overall false positive rate [52]. We chose rather strict criteria to ensure reliability of the results; they can be set differently by other users.

**Figure 1:**
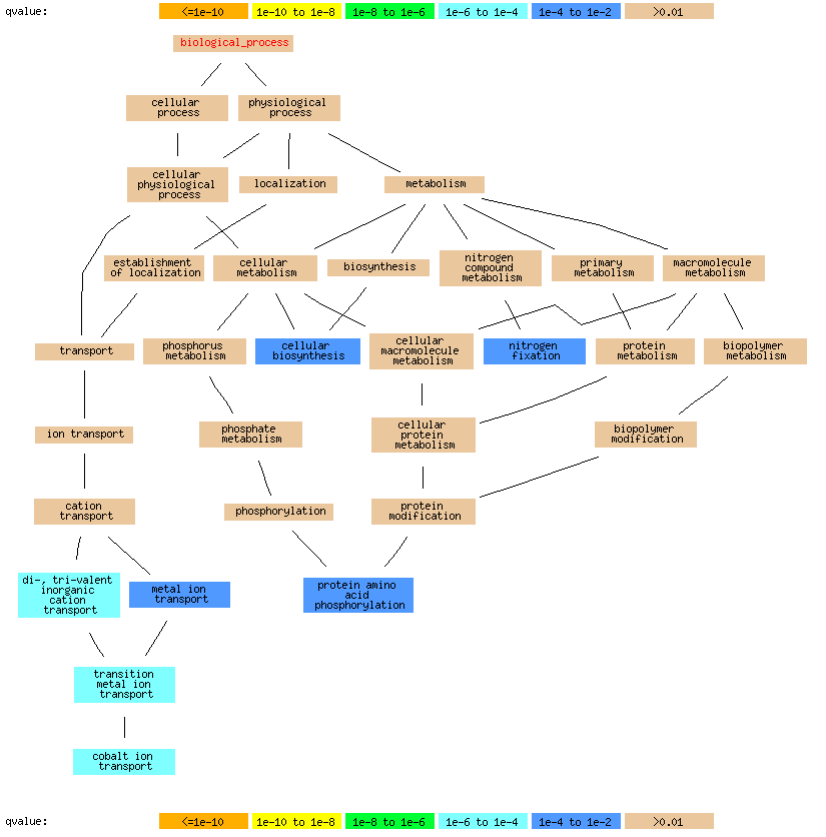
**Comparison of PCC6803 and PCC7120 using our statistical approach**. Comparison of PCC6803 and PCC7120 in the biological process category of GO, using the chi-squared test followed by FDR correction, with the *q*-value cutoff set to 0.01. The colors denote levels of statistical significance of differences between genomes, with the non-significant parent nodes of significant child nodes shown in tan color. (Results for the Molecular Function and Cellular Component categories are available in the Additional file 1)

We found seven terms in the GO Biological Process category that were statistically significantly different between the two genomes, including "transition metal ion transport" (GO:0000041, *q*-value 6.1E-6), "di-, trivalent inorganic cation transport" (GO:0015674, *q*-value 6.1E-6), "cobalt ion transport" (GO:0006824, *q*-value 7.3E-05), "metal ion transport" (GO:0030001, *q*-value 0.00056,), "protein amino acid phosphorylation" (GO:0006468, *q*-value 0.0021), "cellular biosynthesis" (GO:0044249, *q*-value 0.0022) and "nitrogen fixation" (GO:0009399, *q*-value 0.0094). These differences are shown in Figure 1 and discussed below. (The differences detected in the Molecular Function and Cellular Component categories are available in the Additional file 1)

The PCC7120 genome contains significantly more genes in "cobalt ion transport" (GO:0006824) compared with PCC6803, likely a consequence of the multicellular nature of PCC7120. Close inspection showed that the statistically significant difference in parent nodes "transition metal ion transport" (GO:0000041), "di-, trivalent inorganic cation transport" (GO:0015674), and "metal ion transport" (GO:0030001) is a consequence of the difference in the subfamily "cobalt ion transport" (GO:0006824) rather than a cumulative effect of any other subfamilies. PCC7120 contains significantly more genes than PCC6803 in "protein amino acid phosphorylation" (GO:0006468). These genes are responsible for critical protein kinase functions in the multicellular PCC7120 [53-55]. The significantly greater number of genes in "nitrogen fixation" (GO:0009399) in PCC7120 is consistent with its ability to fix nitrogen, a function the simpler organism PCC6803 does not have. The "cellular biosynthesis" (GO:0044249) family differs from those above in that it is significantly more abundant in PCC6803 than in PCC7120. This result may be a consequence of PCC6803's rapid growth capability.

We compared the two genomes with regard to the GO molecular function category and obtained similar results. We then compared them with regard to the GO cellular component category and found three statistically significant differences: "cytoplasm" (GO:0005737), "integral to membrane" (GO:0016021), and "intrinsic to membrane" (GO:0031224), all of which are more abundant in PCC6803 than in PCC7120.

**Figure 2:**
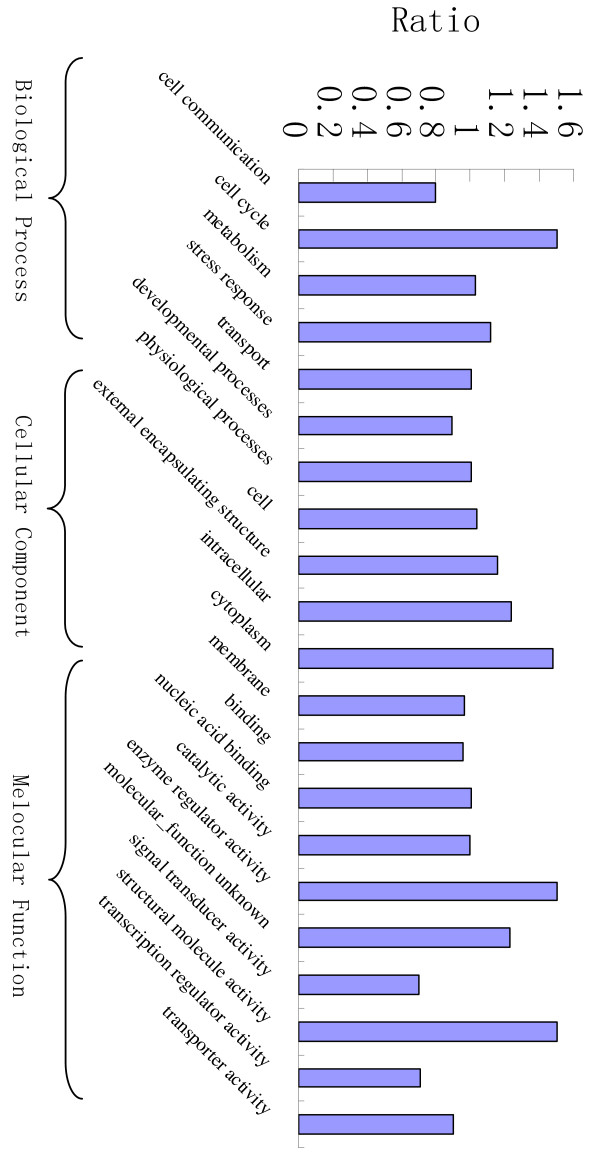
**GOslim-based weighted comparison of PCC6803 and PCC7120**. The bars show the fold difference between PCC6803 and PCC7120 in each GOslim functional catalogory, calculated by the weighted number of genes belonging to the functional category in PCC6803 divided by that in PCC7120. If there is no difference, the fold difference is equal to 1.

We compared our results with results from traditional GO-slim-based, weighted comparison. As shown in Figure 2, the fold difference in the GO-slim-based comparison ranged from 0.7 to 1.5. The fold difference gave only a rough indication of how much PCC6803 and PCC7120 differ in each high-level functional category. In addition, GO-slim-based approach compares two genomes at only the high level, as opposed to our approach that compares at every level and every node. Many important functional differences between two genomes may be detectable only at a finer level. For instance, GO-slim-based approach found little difference between the two cyanobacteria for GOslim term "metabolism, GO:0008152" in the Biological Process category (fold difference 1.03), whereas our approach found that the two species differ significantly in the sub-term "nitrogen fixation, GO:0009399", one of the most important known functional differences.

### Effect of different BLAST cutoffs

**Figure 3:**
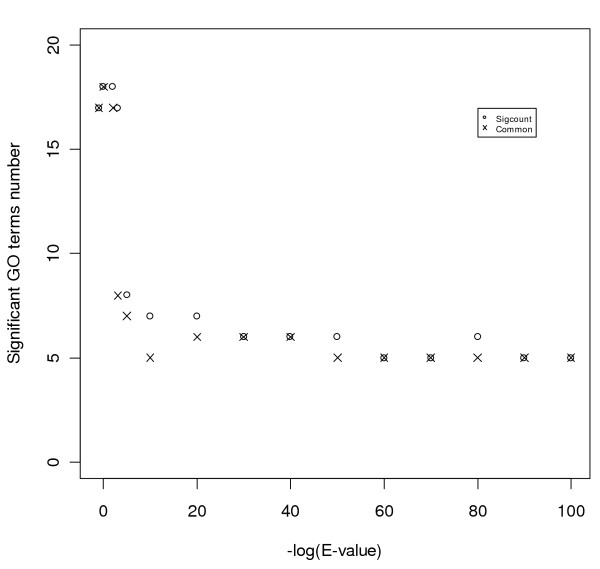
**Effect of different BLAST cutoffs on GO results**. This figure illustrates how much the result changes when the BLAST cutoff is changed. Circles show the number of significant GO terms ("Sigcount") at each cutoff. The symbol '× ' indicates the number of significant GO terms common between a given cutoff and its nearest right neighbor. The BLAST cutoff values range from 1E-100 to 10 (1E-100, 1E-90, 1E-80, 1E-70, 1E-60, 1E-50, 1E-40, 1E-30, 1E-20, 1E-10, 1E-5, 0.001, 0.01, 1, 10). The results stabilize around cutoff values of 1E-20 to 1E-40. We chose a default cutoff of 1E-20.

We varied the BLAST E-value cutoff to study its effect on the number of statistically significant terms detected as well as the number of common terms between adjacent cutoffs. As shown in Figure 3, when the E-value cutoff is high (i.e., less strict, on the left end of the plot), the result is sensitive to the change in cutoff. The results stabilize around cutoff values of 1E-20 to 1E-40. We chose a default cutoff of 1E-20, which coincides with that chosen by GOblet [56].

### Effect of partial data

**Figure 4:**
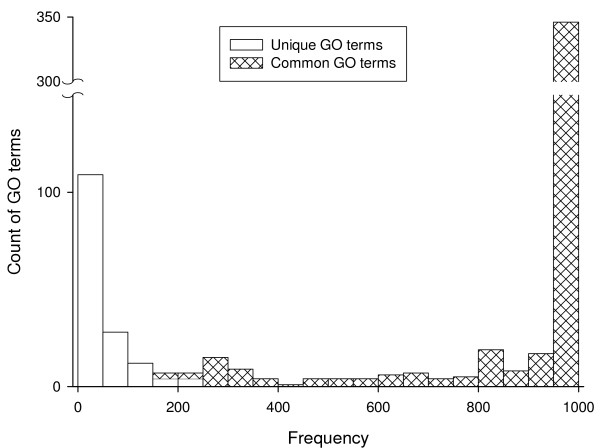
**Histogram of sampling analysis results of the comparison between human and mouse genomes**. The x-axis shows the number of samplings containing a significant GO term, grouped by 50. The y-axis shows the number of terms. The "Common GO terms" are those that occur both in the results from the complete data sets and in at least one sampling. The "Unique GO terms" are those that occur in the result from one or more samplings, but not in the results from the whole data set. For example, the right-most bar shows that 346 "Common GO terms" occurred in the results from 950 or more samples; the left-most bar shows that 109 "Unique GO terms" occurred in the results from less than fifty samples.

**Figure 5:**
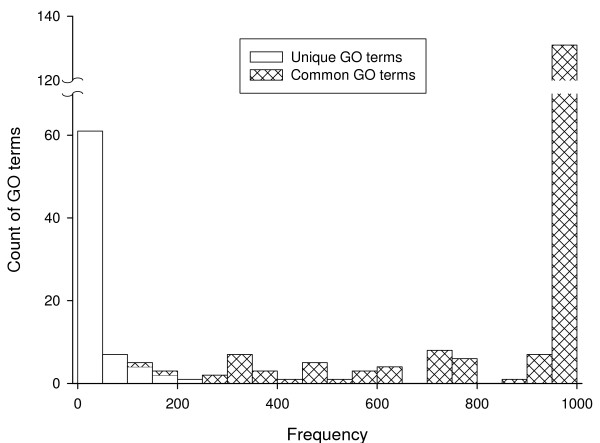
Histogram of sampling analysis results of the comparison between *Saccharomyces cerevisiae *and *Schizosaccharomyces pombe *genomes.

Using the GO-based comparison method, we compared the human and mouse genomes and found 458 statistically significantly different GO terms. We randomly sampled 90% from each of the input gene sets for 1,000 times and compared the statistically significantly different GO terms from each sampling with those from the whole data. As shown in Figure 4 (Hatched bars, "Common GO terms"), most of the GO terms occurred in the majority of the samplings; 298 of the 458 GO terms occurred 1,000 times in all sampling results, whereas at the lower extreme three GO terms occurred only 169 times. This analysis offers an additional measure of reliability of the significant terms detected. The more times a term occurs in the samples, the more reliable it may be. We plotted the distribution of the "unique GO terms"—significant terms detected in one or more of the samples but not in the whole data set—and found that they occurred in as few as one and as many as 247 samples (Figure 4, open bars). As shown in Figure 4, the histogram distributions of the common and unique GO terms overlap slightly. We sampled 60%, 70%, and 80% of the input genes, respectively, and observed similar patterns (see the supplementary figures in the Additional file 1). We performed analysis of the comparison of the two yeast genomes of *Saccharomyces cerevisiae *and *Schizosaccharomyces pombe *and also observed similar patterns (Figure 5 and supplementary figures in the Additional file 1).

## Discussion

BLAST and InterProScan are two most widely used automated GO annotation methods. BLAST is the preferred choice for genome-scale annotation because it runs much faster and, perhaps more importantly, can annotate many more GO terms than InterProScan can. We had used InterProScan to annotate and compare PCC6803 and PCC7120, and found that it missed some important differences including "nitrogen fixation, GO:0009399". However, BLAST has its own limitations. Accurate functional assignment is difficult in cases where the match is less well defined due to lower sequence similarity [57]. In future research we will investigate how to combine results from BLAST and InterProScan to improve annotation quality and use grid computing to reduce computation time.

We used BLAST E-value cutoff as the criteria in assigning GO terms. Local sequence alignment programs such as BLAST may prefer short strong matches to long weak matches and may cause inaccurate GO assignment. The strict E-value cutoff we chose in our analysis ensured the relatively high quality of the results. It was reported that a match between two sequences is most likely reliable if the alignment is at least 70 residues in length with at least 40% sequence identity [58]. We investigated the quality of the HSP (High scoring Segment Pair) in our BLAST results (detail provided in the Additional file 1). With E-value cutoff 1e-20, the minimum length of HSP was 64 and the minimum sequence identity was 68%. Thus the assignments in our results were reliable. It is possible that false negatives may occur with a strict cutoff. In our analysis we prefer accuracy to coverage. Others can use different criteria depending on their individual goals. The statistical testing we proposed in this paper is independent of the GO assignment method. We suggest doing the comparison and comparing the results using different E-value cutoffs and different subsets of the input gene sets to identify the most reliable differences between two genomes.

In any GO analysis, the quality of the original GO annotation is critical. The GO annotation data are continuously expanded; however, the present data are incomplete and noisy [59], and the annotation quality is uneven, with a mix of literature-supported annotations and those inferred automatically. We did not modify the GO annotation data for our present study, but further research will consider the quality of the original GO annotations when assessing the reliability of the results. One limitation of our approach is that it only compared the number of genes in each functional category. It cannot capture differences in the level of gene expression. Another inherent limitation of GO is that it does not map directly to pathways. As a result GO-based comparison cannot detect differences at the pathway level. We have recently used the KEGG Orthology (KO) as an alternative controlled vocabulary in a KO-Based Annotation System (KOBAS) and demonstrated that KOBAS is effective in automated annotation and pathway identification [60]. In future research we will investigate KO-based comparison to compare two genomes at the pathway level.

Our goal is to achieve higher confidence in the differences detected between two genomes. Towards this end, we applied rigorous statistical testing followed by FDR correction instead of simply relying on fold changes. We also tested a wide range of BLAST cutoff values and different subsets of the input genes to provide additional measures of confidence in the results. If results beyond those having the highest confidence are required, then the cutoff values can be relaxed. The advantage of the statistical approach presented here is that, no matter what cutoff values are chosen, the resulting *p*-values, *q*-values, and sampling analysis can be used to assess the confidence in the results.

There are other procedures available to correct false positive rates resulting from multiple testing, including the Bonferroni correction, Sidak stepwise correction, Holm stepwise correction, Hochberg's stepwise correction, and others [61,62]. We chose the FDR correction because of its overall high quality and computational speed [63,64]. It is also the most common procedure used in GO-related and microarray analyses [62,65,66].

## Conclusion

Contrary to the situation in microarray analysis, there is a surprising lack of statistical approaches used in GO-based comparison of two complete genomes. Our work is the first to propose and test a statistical approach to comparing the complete sets of genes in two whole genomes at all levels of GO and study the effect of varying BLAST cutoffs and using subset of the input gene sets. We believe that such an approach can provide a measure of confidence in the identified differences and help ensure the reliability of the results.

## Methods

Supplementary materials and related programs for the paper are provided on-line [See Additional file 1].

### Whole-genome GO annotation

We set the default BLAST cutoff E-value to be 1E-20. In Part 3 of results, we study the cutoff's effect on the final results. We parsed the BLAST result to obtain the GOA ID for the top hit and used the ID to query the GOA association database to retrieve the corresponding GO annotation and assign it to the query sequence. The result is written to a file in the format specified by the GO Consortium [67].

We parsed the gene ontology DAGs and stored the GO terms and their hierarchical relationships in a local data structure. The genes in a genome are linked to GO terms using the aforementioned approach; they are also linked to all parent GO terms by propagating the DAG structures. If a gene has been assigned more than one GO terms that have a common parent GO term, the gene is counted only once in the parent GO term. Finally, the numbers of genes assigned to each GO term in the DAGs are tallied, representing the abundance of genes in each GO function within the genome.

The complete set of known and predicted genes in PCC6803 and PCC7120 genomes were downloaded from Cyanobase [68]. The PCC6803 genome contains 3,573,470 bp with 3,167 predicted ORFs; the PCC7120 genome contains 6,413,771 bp with 5,362 predicted ORFs.

### Testing the statistical significance of detected differences between genomes

The goal is to identify all GO terms for which two genomes (A and B) are statistically significantly different. Define:

N = the total number of annotated genes in Genome A

n = the total number of annotated genes in Genome B

X = the number of genes in Genome A that are assigned the GO term currently under consideration

x = the number of genes in Genome B that are assigned the GO term currently under consideration

We used the chi-squared test to address whether the ratios,  and , come from the same distribution, either:

H_0_: ***p*_0 _**= ***p*_1 _**or

H_1_: ***p*_0 _**≠ ***p*_1_**

The *p-*value is calculated as the upper tail probability of the chi-squared distribution with one degree of freedom using the CPAN Statistics::Distributions modules [69].

Because the number of tests performed equals the number of GO terms, which may be thousands, multiple hypotheses testing is important to control the overall Type I error rate. We used the commonly applied FDR correction. For every test result that is considered statistically significant, the FDR correction calculates a *q-*value to measure the minimum FDR when calling that result significant. A *q-*value cutoff, α (alpha), guarantees that the expected proportion of false positives is α (alpha) among the set of significant features produced [52,66]. The default for α (alpha) was set to 0.01 in our study. The conservative FDR correction was implemented according to the GenTS package [70].

The statistically significantly different GO terms detected between two genomes are stored in text format, sorted by increasing *q*-value. We also modified the GO TermFinder package [71] to show the results graphically, with different colors showing different levels of significance.

All related programs are attached in Additional file 1

### Effect of different BLAST cutoffs

We studied how the BLAST cutoff value can affect the comparison of results between two genomes of PCC6803 and PCC7120. We tested a wide range of BLAST E-value cutoffs, from 1E-100 to 10, and recorded the number of statistically significantly different GO terms between the two cyanobacterial genomes at each cutoff. We then recorded the number of common statistically significantly different GO terms between adjacent cutoffs to show how much the result changes when the cutoff is varied.

### Effect of partial data

We performed the random sampling to study how the results are affected when only part of the data is used. For each sample, we randomly selected 90%, 80%, 70%, and 60% of the annotated genes in each genome, and recomputed the statistically significantly different GO terms. We then compared the result of each sampling with that for the complete data sets and counted the numbers of common and unique GO terms. Because comparison of the two cyanobacteria resulted in too few significant GO terms to make this analysis meaningful, we analyzed the comparison of human *vs*. mouse and *Saccharomyces cerevisiae vs. Schizosaccharomyces pombe*. The GO annotations for these four genomes were retrieved from the Gene Ontology Consortium web site.

## Authors' contributions

ZC and LW conceived of the study; ZC carried out most of the implementation and analysis; XM and LW participated in the analysis; SL participated in the design of statistical tests. All authors participated in preparation of the manuscript.

## Supplementary Material

Additional File 1 **Supplementary materials and related programs**. The compressed file contains supplementary materials and related programs for the paper, including the source codes and documents, the genome comparison results between PCC6803_PCC7120, Cerevisiae_Pombe and Human_Mouse, the figures for the effect of using different subsets of the input genes and the statistical analysis about the BLAST HSP (High scoring Segment Pair) length. Please unzip the file and read the "index.htm" for detail. Also, you can visit the website for the information (). Click here for file

## Figures and Tables

**Table 1:** Comparison of AMIGO and GOA

GO Annotation Database	AMIGO^1^	GOA^2^
Curator	GO Consortium	European Bioinformatics Institute (EBI)
URL		
Total number of species	129,722	96,203
Total number of associations	7,745,168	7,600,805
Total number of non-redundant sequences	219,341	1,605,096
Total number of GO terms	10,916	9,258
Total number of other databases integrated	143	14

^1^AMIGO monthly release (November 1, 2005) downloaded from

^2^GOA version 33.0 (October 25, 2005) downloaded from
