# Supplementary material for: Genome comparison using Gene Ontology (GO) with statistical testing
Source: BMC Bioinformatics. 2006 Aug 11;7:374. doi: 10.1186/1471-2105-7-374 (PMC1569881; doi:10.1186/1471-2105-7-374)
Supplement: Additional File 1 — Supplementary materials and related programs. The compressed file contains supplementary materials and related programs for the paper, including the source codes and documents, the genome comparison results between PCC6803_PCC7120, Cerevisiae_Pombe and Human_Mouse, the figures for the effect of using different subsets of the input genes and the statistical analysis about the BLAST HSP (High scoring Segment Pair) length. Please unzip the file and read the "index.htm" for detail. Also, you can visit the website for the information (). [file 1471-2105-7-374-S1.zip › GO/demo.htm]

demo


# Demo

---

- ## **GO Annotation file**

  We have complied with the file format described by the GeneOntology consortium
    
  for annotation files (http://www.geneontology.org/GO.annotation.html#file).
  Since we provide the GO annotation based on sequence similarity, some fields
  are filled with space symbol.  
  The examples of goa file can be download from http://www.geneontology.org/GO.current.annotations.shtml

  - ## **Background file**

    The background file format is like this:

    The first line gives the total annotated gene number. Then for every GO term,
    the file gives gene number contained in the GO term and the proportion of
    the gene number to the total annotated gene number.

    - ## **Qvalue file**

      The qvalue file format is like this:

      The first column is the GO term,  
      The second column is the corrected Pvalue with conservative FDR method,  
      The third column is the original Pvalue based on Chi-squared test.  
      The fourth column is the GO name,  
      The fifth column is the GO aspect, one of process ontology (P), component
      ontolgoy (C), function ontology (F);

      Then, the following several columns come from the background files which
      produce the pvalue file. We list the background file name, the gene total
      number contained in the GO term and the proportion of the gene number to the
      total annotated gene number.
