# Supplementary material for: Genome comparison using Gene Ontology (GO) with statistical testing
Source: BMC Bioinformatics. 2006 Aug 11;7:374. doi: 10.1186/1471-2105-7-374 (PMC1569881; doi:10.1186/1471-2105-7-374)
Supplement: Additional File 1 — Supplementary materials and related programs. The compressed file contains supplementary materials and related programs for the paper, including the source codes and documents, the genome comparison results between PCC6803_PCC7120, Cerevisiae_Pombe and Human_Mouse, the figures for the effect of using different subsets of the input genes and the statistical analysis about the BLAST HSP (High scoring Segment Pair) length. Please unzip the file and read the "index.htm" for detail. Also, you can visit the website for the information (). [file 1471-2105-7-374-S1.zip › GO/index.htm]

index


# Supplemental materials of

# "Genome Comparison Using Gene Ontology (GO) with Statistical Testing"

---

The PCC6803\_PCC7120 comparison results:

Qvalue (Excel File), GOview
(Excel File)

The Cerevisiae\_Pombe comparison results:

Qvalue (Excel File), GOview
(Excel File)

The Human\_Mouse comparison results:

Qvalue (Excel File), GOview
(Excel File)

---

We provided the related programs. The package is demo version now. Please read
the manual firstly.

Manual

Perl scripts (4.6M)

GO-termfinder modules (875K)

GOA related file:

fasta sequence file (Please download from our ftp server ftp://ftp.cbi.pku.edu.cn/pub/cbird/GO/)

association file (Please download the latest version from EBI ftp server ftp://ftp.ebi.ac.uk/pub/databases/GO/goa/UNIPROT/gene\_association.goa\_uniprot.gz).

---

HSP distribution in BLAST resutls

With cutoff evlaue 1e-20, the HSP length (aa) summary

|  |  |  |  |  |  |
| --- | --- | --- | --- | --- | --- |
| Min | 1st Qu. | Median | Mean | 3rd Qu. | Max. |
| 64 | 257 | 386 | 470.5 | 550.8 | 4826 |

The min HSP length is 64, percent identity is 68%

With cutoff evlaue 1e-40, the HSP length (aa) summary

|  |  |  |  |  |  |
| --- | --- | --- | --- | --- | --- |
| Min | 1st Qu. | Median | Mean | 3rd Qu. | Max. |
| 92 | 309 | 436 | 526.3 | 610.8 | 4826 |

The min HSP length is 92, percent identity is 85%

---

Effect of partial data sampling:

Human\_Mouse comparison 60%, 70%, 80%, 90% sampling:

The Cerevisiae\_Pombe comparison 60%, 70%, 80%, 90% sampling:

---
