# Supplementary material for: Genome comparison using Gene Ontology (GO) with statistical testing
Source: BMC Bioinformatics. 2006 Aug 11;7:374. doi: 10.1186/1471-2105-7-374 (PMC1569881; doi:10.1186/1471-2105-7-374)
Supplement: Additional File 1 — Supplementary materials and related programs. The compressed file contains supplementary materials and related programs for the paper, including the source codes and documents, the genome comparison results between PCC6803_PCC7120, Cerevisiae_Pombe and Human_Mouse, the figures for the effect of using different subsets of the input genes and the statistical analysis about the BLAST HSP (High scoring Segment Pair) length. Please unzip the file and read the "index.htm" for detail. Also, you can visit the website for the information (). [file 1471-2105-7-374-S1.zip › GO/manual.htm]

manual


# README

The package is developed for the genome comparison using GO. We utilized the
Blast tool for genome level GO electronic annotation. The subject database used
in our method is GOA database (We checked out the sequences from SwissProt,TrEMBL
and Uniref sequences databases basing on the DB\_Object\_ID field of GOA database).
After the GO annotation, for each GO term the abundance of genes in the two
genomes is compared using a chi-squared test followed by false discovery rate
(FDR) correction.

---

## Install the required environment

- NCBI BLAST2 Standalone Edition;  
  - MySQL server (version 3.23.58 or later)  
    - Perl-DBI (version 1.40 or later)  
      - BioPerl (version 1.4 or later)  
        - GO-TermFinder-0.61 (If you want to draw the GO DAG picture, please also install
          the Perl-GD, Perl-GraphViz modules, see README in TermFinder)  
          - Statistics-Distributions (version 1.02 or later)  

            The simple method to check whether you had installed above-mentioned perl
            modules, please try:  
            >perldoc bioperl  
            >perldoc DBI  
            >perldoc GO  
            >perldoc Statistics::Distributions

            We provided the GO-termfinder package in the website. "GO-TermFinder"
            is the workhorse used to parse the ontology DAG structure and draw the picture.
            To install it, please refer to "README" in the package. This module
            is revised for our purpose.   
            Generally, the procedure of installing Perl modules is:  
            >perl Makefile.PL  
            >make   
            >make test  
            >make install

            ## Create the GOA database

            >perl createdb.pl   
            # You need the MySQL server root privileges, please change the script for
            your purpose.  
            >perl load\_goa.pl  
            # You at least leave 1G space for the "goa" table in MySQL.   
            >formatdb -i goaseq.fasta -n goaseq   
            # use BLAST "formatdb" command to index the GOA fasta seqences.

            ## Comparative genome pipeline using GO

            1. Run blastp|x  
            >blastall -p blastp|x -i genomeseq.fasta -d goaseq > genomeseq.bls;

            2. Parse blastp result  
            >perl parseblast.pl genomeseq.bls cutoffevalue(default = 10) > genomeseq.bls.parsed;

            3. GO annotation.  
            >perl goassocciation.pl genomeseq.bls.parsed
            > genomeseq.goa;

            4. GOslim Analysis  
            >perl goslim.pl genomeseq.goa > genomeseq.goslim;

            5. Calculate the background distribution  
            >perl background.pl genomeseq.goa > backgroundgenome.txt;

            6. View the background distribution  
            >perl backgroundview.pl [P|F|C] backgroundgenome.txt
            imagename countcutoff [default 100];

            7. Calculate the pvalue  
            >perl pvalue\_chisq.pl [P|F|C] backgroundgenome1.txt
            backgroundgenome2.txt ... > pvaluefile.txt;

            8. Print the comparison in text format.  
            >perl goviewtxt.pl [P|F|C] pvaluefile.txt qvaluecutoff(default
            0.01) > goview.txt;

            9. Output the PNG picture  
            >perl goview.pl [P|F|C] pvaluefile.txt imagename
            qvaluecutoff(default 0.01);

            ## Program description

            goassociation.pl: Produce the GO association file: | | | --- | | Generally it needs 3 steps for the GO association. First, runs BlastP|X with your provided protein or cDNA sequences; then, parses the Blast results, at last, links the GO to your query sequences using the Blast search results. The output results are complied with the file format described by the GeneOntology consortium for annotation files (description). To run the program, you will need the right to create a table in MySQL database. | goslim.pl: GOslim analysis: | | | --- | | The analysis uses the GOA GOslim flat file "goslim\_goa.goa"(http://www.geneontology.org/GO\_slims/goslim\_goa.go). Also, you can change the variable of the program for your purpose. You need to provide the GO association file (the GOA consistent file format). The results will be one tab-split text file: goslim\_out.txt. | background.pl: Analyze the genes distribution in the whole level of GO DAG.: | | | --- | | In the GObackground analysis, The genes linked to one GO term also are linked to all its ancestor terms depending on the GO relations in GO DAG (Directed Acyclic Graph). You need to provide the GO association file (the GOA consistent file format) for the analysis. The result will be a background format file (description): backgroundgo.txt | backgroundview.pl: Display the genes distribution in the picture: | | | --- | | You need select one of aspects (P, biological process; F, molecular function; C, cell component) and the count cutoff (control to display the GO terms containing gene number more than the cutoff value). And the result will be a PNG format picture: backgroundview.png You can change the configure file: "GoView.conf" for your purpose | pvalue\_chisq.pl: Find the significantly different GO terms: | | | --- | | You need to choose one of aspects (P|F|C) and provide two or more background files to compare. The result will be a Pvalue format file (description): pvalue\_chisquare.txt. | goview.pl: View the significant GO terms in the picture: | | | --- | | You need to choose one of aspects (P|F|C) and provide the pvalue file (the aspect of pvalue file should be consistent with your chose aspect). You can control to display which GO nodes by setting qvalue cutoff (default 0.01). The result will be a PNG file: goview.png. You can change the configure file: "GoView.conf" for your purpose. | goviewtxt.pl: View the significant GO terms in the text format: | | | --- | | It is similar with goview.pl. But it can give more detail information in the result file: goview.txt. |
