# Supplementary figures and images for: Genome comparison using Gene Ontology (GO) with statistical testing
Source: BMC Bioinformatics. 2006 Aug 11;7:374. doi: 10.1186/1471-2105-7-374 (PMC1569881; doi:10.1186/1471-2105-7-374)

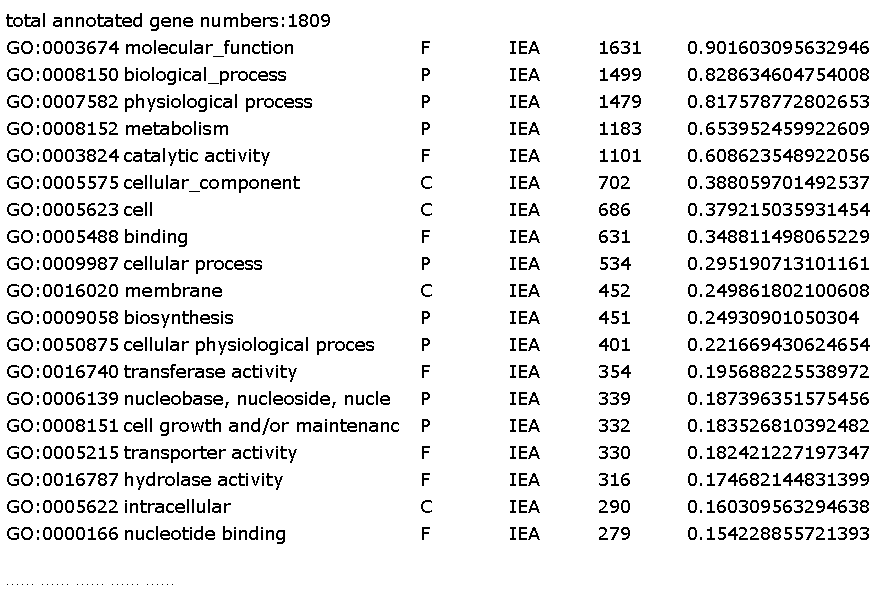

Supplement: Additional File 1 — Supplementary materials and related programs. The compressed file contains supplementary materials and related programs for the paper, including the source codes and documents, the genome comparison results between PCC6803_PCC7120, Cerevisiae_Pombe and Human_Mouse, the figures for the effect of using different subsets of the input genes and the statistical analysis about the BLAST HSP (High scoring Segment Pair) length. Please unzip the file and read the "index.htm" for detail. Also, you can visit the website for the information (). [file 1471-2105-7-374-S1.zip › GO/bg.png]

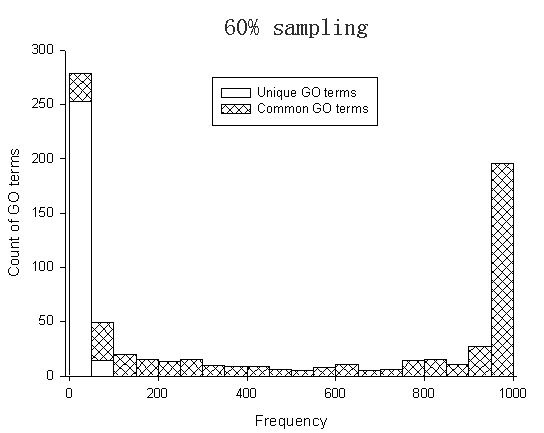

Supplement: Additional File 1 — Supplementary materials and related programs. The compressed file contains supplementary materials and related programs for the paper, including the source codes and documents, the genome comparison results between PCC6803_PCC7120, Cerevisiae_Pombe and Human_Mouse, the figures for the effect of using different subsets of the input genes and the statistical analysis about the BLAST HSP (High scoring Segment Pair) length. Please unzip the file and read the "index.htm" for detail. Also, you can visit the website for the information (). [file 1471-2105-7-374-S1.zip › GO/human_mouse_60.gif]

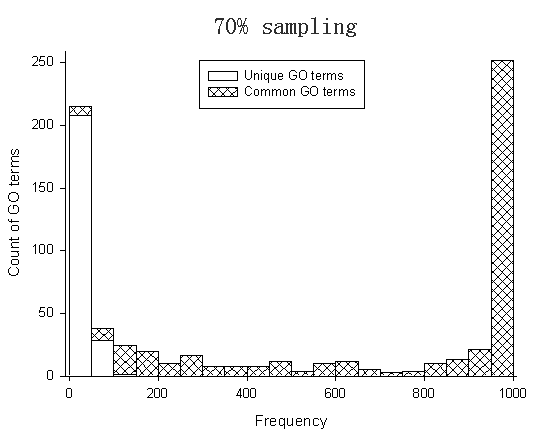

Supplement: Additional File 1 — Supplementary materials and related programs. The compressed file contains supplementary materials and related programs for the paper, including the source codes and documents, the genome comparison results between PCC6803_PCC7120, Cerevisiae_Pombe and Human_Mouse, the figures for the effect of using different subsets of the input genes and the statistical analysis about the BLAST HSP (High scoring Segment Pair) length. Please unzip the file and read the "index.htm" for detail. Also, you can visit the website for the information (). [file 1471-2105-7-374-S1.zip › GO/human_mouse_70.gif]

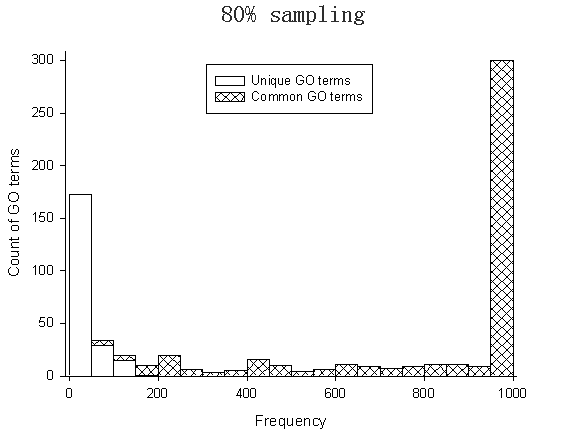

Supplement: Additional File 1 — Supplementary materials and related programs. The compressed file contains supplementary materials and related programs for the paper, including the source codes and documents, the genome comparison results between PCC6803_PCC7120, Cerevisiae_Pombe and Human_Mouse, the figures for the effect of using different subsets of the input genes and the statistical analysis about the BLAST HSP (High scoring Segment Pair) length. Please unzip the file and read the "index.htm" for detail. Also, you can visit the website for the information (). [file 1471-2105-7-374-S1.zip › GO/human_mouse_80.gif]

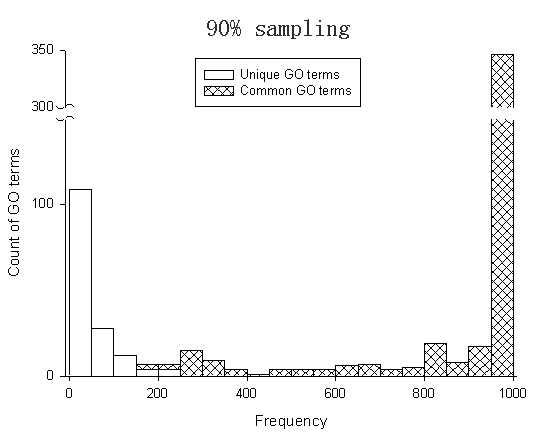

Supplement: Additional File 1 — Supplementary materials and related programs. The compressed file contains supplementary materials and related programs for the paper, including the source codes and documents, the genome comparison results between PCC6803_PCC7120, Cerevisiae_Pombe and Human_Mouse, the figures for the effect of using different subsets of the input genes and the statistical analysis about the BLAST HSP (High scoring Segment Pair) length. Please unzip the file and read the "index.htm" for detail. Also, you can visit the website for the information (). [file 1471-2105-7-374-S1.zip › GO/human_mouse_90.gif]

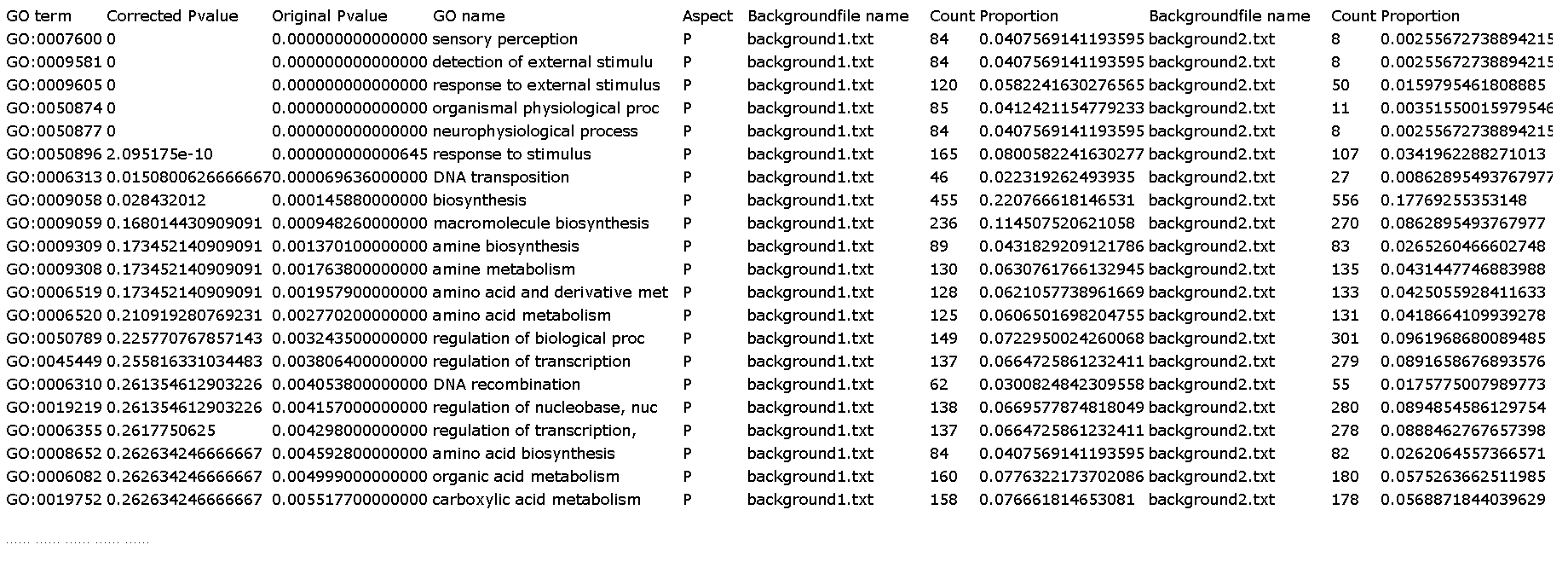

Supplement: Additional File 1 — Supplementary materials and related programs. The compressed file contains supplementary materials and related programs for the paper, including the source codes and documents, the genome comparison results between PCC6803_PCC7120, Cerevisiae_Pombe and Human_Mouse, the figures for the effect of using different subsets of the input genes and the statistical analysis about the BLAST HSP (High scoring Segment Pair) length. Please unzip the file and read the "index.htm" for detail. Also, you can visit the website for the information (). [file 1471-2105-7-374-S1.zip › GO/pvalue.png]

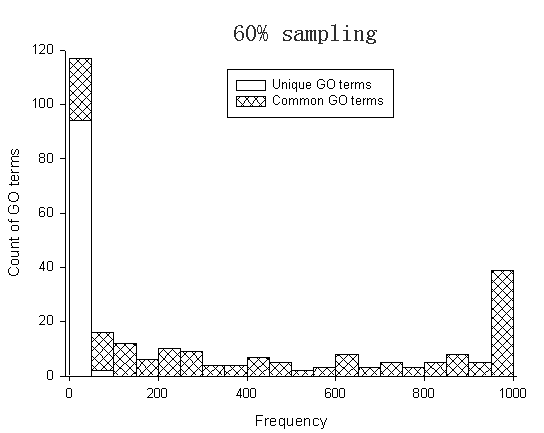

Supplement: Additional File 1 — Supplementary materials and related programs. The compressed file contains supplementary materials and related programs for the paper, including the source codes and documents, the genome comparison results between PCC6803_PCC7120, Cerevisiae_Pombe and Human_Mouse, the figures for the effect of using different subsets of the input genes and the statistical analysis about the BLAST HSP (High scoring Segment Pair) length. Please unzip the file and read the "index.htm" for detail. Also, you can visit the website for the information (). [file 1471-2105-7-374-S1.zip › GO/yeasts_60.gif]

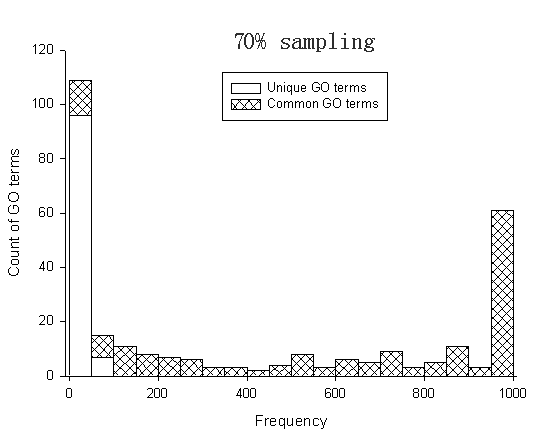

Supplement: Additional File 1 — Supplementary materials and related programs. The compressed file contains supplementary materials and related programs for the paper, including the source codes and documents, the genome comparison results between PCC6803_PCC7120, Cerevisiae_Pombe and Human_Mouse, the figures for the effect of using different subsets of the input genes and the statistical analysis about the BLAST HSP (High scoring Segment Pair) length. Please unzip the file and read the "index.htm" for detail. Also, you can visit the website for the information (). [file 1471-2105-7-374-S1.zip › GO/yeasts_70.gif]

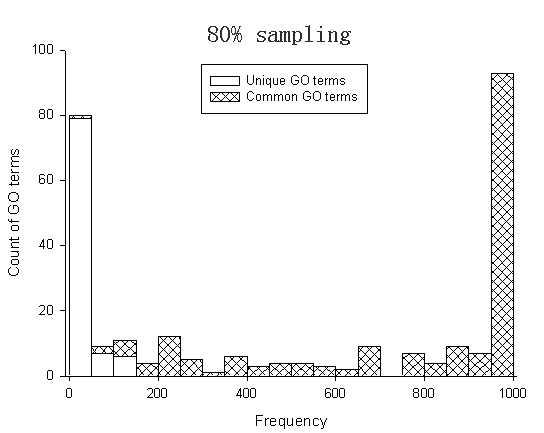

Supplement: Additional File 1 — Supplementary materials and related programs. The compressed file contains supplementary materials and related programs for the paper, including the source codes and documents, the genome comparison results between PCC6803_PCC7120, Cerevisiae_Pombe and Human_Mouse, the figures for the effect of using different subsets of the input genes and the statistical analysis about the BLAST HSP (High scoring Segment Pair) length. Please unzip the file and read the "index.htm" for detail. Also, you can visit the website for the information (). [file 1471-2105-7-374-S1.zip › GO/yeasts_80.gif]

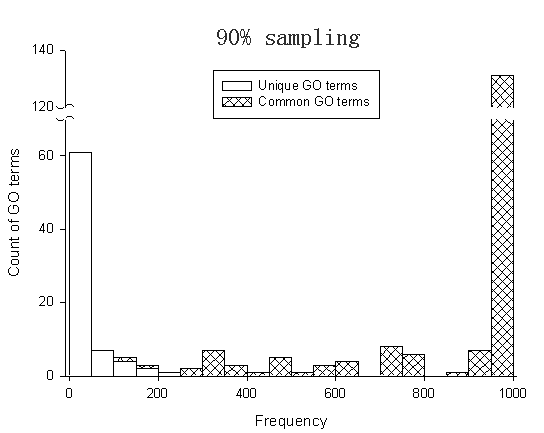

Supplement: Additional File 1 — Supplementary materials and related programs. The compressed file contains supplementary materials and related programs for the paper, including the source codes and documents, the genome comparison results between PCC6803_PCC7120, Cerevisiae_Pombe and Human_Mouse, the figures for the effect of using different subsets of the input genes and the statistical analysis about the BLAST HSP (High scoring Segment Pair) length. Please unzip the file and read the "index.htm" for detail. Also, you can visit the website for the information (). [file 1471-2105-7-374-S1.zip › GO/yeasts_90.gif]
