# Supplementary material for: Genome comparison using Gene Ontology (GO) with statistical testing
Source: BMC Bioinformatics. 2006 Aug 11;7:374. doi: 10.1186/1471-2105-7-374 (PMC1569881; doi:10.1186/1471-2105-7-374)
Supplement: Additional File 1 — Supplementary materials and related programs. The compressed file contains supplementary materials and related programs for the paper, including the source codes and documents, the genome comparison results between PCC6803_PCC7120, Cerevisiae_Pombe and Human_Mouse, the figures for the effect of using different subsets of the input genes and the statistical analysis about the BLAST HSP (High scoring Segment Pair) length. Please unzip the file and read the "index.htm" for detail. Also, you can visit the website for the information (). [file 1471-2105-7-374-S1.zip › GO/termfinder.tar.gz!/GO-TermFinder-0.61/examples/examples.html]

Example Clients of GO::TermFinder and related modules


# Example Clients of GO::TermFinder and related modules


This directory contains various example client scripts that can be
used to access the functionality of of the GO::TermFinder and related
modules, that provide a rich API for accessing information from the
Gene Ontology project. These examples serve the purpose of providing
useful example code for people who wish to write alternate clients of
these objects, as well as providing useful functionality in their own
right.

Before running any of the example programs, you should first install
the GO::TermFinder modules, according to the instructions in the
README file. Also, see the associated pod for each example program
for more information about that particular program.

The example client programs are:

1. analyze.pl

This program takes a list of files, each of which contain a list of
genes, with one gene per line. It will findTerms for the lists of
genes in each of the GO aspects, outputting the results to a file
named for the original file, but with a .terms extension. It will
only output terms with a corrected P-value of <= 0.05.
It will use the first supplied argument as the annotation file, the
second argument as the expected number of genes within the organism,
and all subsequent files as ones containing lists of genes. You need
to provide the ontology files in the same directory from which you are
executing this script, and the GO-TermFinder libraries must be in your
path.

```
Usage:

    analyze.pl <annotation_file> <numGenes> <file1> <file2> <file3> ... <fileN>

e.g.

    analyze.pl gene_association.sgd 7200 file1.txt file2.txt file3.txt
```

2. ancestors.pl

ancestors.pl simply takes as input a GOID, and an ontology file, and prints out
the all the paths from that GO node to the root of the ontology, e.g:

```
    >ancestors.pl GO:0008346 ../t/process.ontology
    GO:0003673    Gene_Ontology
        GO:0008150    biological_process
            GO:0007610    behavior
                GO:0030537    larval behavior
                    GO:0008345    larval locomotory behavior
                        GO:0008346    larval walking behavior

    GO:0003673    Gene_Ontology
        GO:0008150    biological_process
            GO:0007610    behavior
                GO:0007626    locomotory behavior
                    GO:0008345    larval locomotory behavior
                        GO:0008346    larval walking behavior
```

3. batchGOView.pl

batchGoView.pl will read through a number of files, each containing a
list of genes, and will create for each one an html page with a
GO::View, such that you can graphically browse the results. You need
to provide a .conf file, and then a list of files for processing, each
of which contain a list of genes. An example .conf file exists in
this directory - edit as appropriate. The following usage should give
you some output, using the example files.
Usage:
batchGOView.pl <.conf\_file>> <file1>> <file2> <file3> ... <fileN>
e.g.
batchGOView.pl GoView.conf genes.txt genes2.txt

Notes about the conf file:  
  
The .conf file is simply a file that describes various parameters that
can be used by batchGOView.pl. Each parameter is in the form of:

```
name = value
```

lines beginning with # are comment lines. See the example .conf file
in the same directory as this html file.

Output from batchGOView.pl looks something like:

## Terms for genes.txt

---


### Result Table

**Terms from the Process Ontology exceeding a pvalue cutoff of 1e-12**

| Gene Ontology term | Cluster frequency | Genome frequency of use | P-value | Genes annotated to the term |
| --- | --- | --- | --- | --- |
| sulfur metabolism | 13 out of 19 genes, 68.4% | 45 out of 6905 genes, 0.7% | 1.79e-24 | STR3, MHT1, MET17, MET6, ECM17, MET1, MET2, MET3, MET16, MET28, MET11, MET14, MET10 |
| sulfur amino acid metabolism | 11 out of 19 genes, 57.9% | 29 out of 6905 genes, 0.4% | 7.27e-22 | STR3, MHT1, MET17, MET6, MET1, MET2, MET3, MET16, MET28, MET11, MET14 |
| methionine metabolism | 9 out of 19 genes, 47.4% | 20 out of 6905 genes, 0.3% | 1.87e-18 | STR3, MET17, MET6, MET1, MET2, MET3, MET16, MET11, MET14 |
| aspartate family amino acid metabolism | 9 out of 19 genes, 47.4% | 41 out of 6905 genes, 0.6% | 3.80e-15 | STR3, MET17, MET6, MET1, MET2, MET3, MET16, MET11, MET14 |
| sulfate assimilation | 6 out of 19 genes, 31.6% | 8 out of 6905 genes, 0.1% | 6.04e-14 | MET14, ECM17, MET1, MET3, MET16, MET10 |
| sulfur utilization | 6 out of 19 genes, 31.6% | 8 out of 6905 genes, 0.1% | 6.04e-14 | MET14, ECM17, MET1, MET3, MET16, MET10 |
| amino acid metabolism | 11 out of 19 genes, 57.9% | 141 out of 6905 genes, 2.0% | 1.37e-13 | STR3, MHT1, MET17, MET6, MET1, MET2, MET3, MET16, MET28, MET11, MET14 |
| amino acid and derivative metabolism | 11 out of 19 genes, 57.9% | 151 out of 6905 genes, 2.2% | 2.96e-13 | STR3, MHT1, MET17, MET6, MET1, MET2, MET3, MET16, MET28, MET11, MET14 |
| amine metabolism | 11 out of 19 genes, 57.9% | 161 out of 6905 genes, 2.3% | 6.07e-13 | STR3, MHT1, MET17, MET6, MET1, MET2, MET3, MET16, MET28, MET11, MET14 |

- children.pl

children.pl simply takes as input a GOID, and an ontology file, and prints out
the children of that GO node, e.g.:

```
    >children.pl GO:0008150 process.ontology

    Children of GO:0008150 (biological_process) : 

    GO:0000004    biological_process unknown
    GO:0016032    viral life cycle
    GO:0007582    physiological processes
    GO:0007275    development
    GO:0009987    cellular process
    GO:0008371    obsolete
    GO:0007610    behavior
```

- termFinderClient.pl

  This program is a very simply client for the GO::TermFinder object,
  that prompts a user for the various pieces of information that are
  required to determine significant GO terms associated with the list of
  genes. It uses a p-value cut-off (for the corrected p-value) of .001,
  and simply prints the information back to the screen.

  Feedback on any and all of this software, clients or libraries alike,
  is welcomed and encouraged. We are more than happy to implement
  reasonable improvements and changes, and of course we always want to
  fix bugs. Please send feedback to Gavin Sherlock
