# Supplementary material for: Genome comparison using Gene Ontology (GO) with statistical testing
Source: BMC Bioinformatics. 2006 Aug 11;7:374. doi: 10.1186/1471-2105-7-374 (PMC1569881; doi:10.1186/1471-2105-7-374)
Supplement: Additional File 1 — Supplementary materials and related programs. The compressed file contains supplementary materials and related programs for the paper, including the source codes and documents, the genome comparison results between PCC6803_PCC7120, Cerevisiae_Pombe and Human_Mouse, the figures for the effect of using different subsets of the input genes and the statistical analysis about the BLAST HSP (High scoring Segment Pair) length. Please unzip the file and read the "index.htm" for detail. Also, you can visit the website for the information (). [file 1471-2105-7-374-S1.zip › GO/termfinder.tar.gz!/GO-TermFinder-0.61/docs/GO-TermFinder.doc]

# How GO::TermFinder calculates P-values

The GO::TermFinder attempts to determine whether an observed level of annotation for a group of genes is significant within the context of annotation for all genes within the genome. Suppose that we have a total population of N genes, in which M have a particular annotation. If we observe x genes with that annotation, in a sample of n genes, then we can calculate the probability of that observation, using the hypergeometric distribution (e.g., see <http://mathworld.wolfram.com/HypergeometricDistribution.html>) as:

$$p=\begin{aligned} \left( M | \right) \\ \end{aligned}$$

where generically,

$$\begin{aligned} \left( n | \right) \\ \end{aligned}$$

which is the number of permutations by which r entities can be selected from n entities, is calculated as:

$$\frac{n!}{r!(n-r)!}$$

To actually generate a p-value, rather than a simple probability, instead of asking the question, what is the probability of having 5 out of 10 genes with this annotation, given that 42 out 6000 have it, we ask the question what is the probability of having 5 *or more* out of 10 genes having this annotation. This is what a p-value is – the chance of seeing your observation, or better, given the background distribution. We calculate this by summing our probabilities for 5 out of 10, 6 out of 10, 7 out of 10 etc. Thus the probability of seeing x or more genes with an annotation, out n, given that M in the population of N have that annotation, is:

$$p_{\text{value}}=\sum_{j=x}^{n} \begin{aligned} \left( M | \right) \\ \end{aligned}$$

Note that this is the same as saying what’s the chance of getting *at least* x successes, and can also be represented by:

$$p_{\text{value}}\text{ = }1-\sum_{j=0}^{x-1} \begin{aligned} \left( M | \right) \\ \end{aligned}$$

Typically, a cut-off for p-values, known as the alpha level, is chosen, such that p-values below the alpha level are deemed significant. The alpha level is the chance taken by researchers to make a type one error. The type one error is the error of incorrectly declaring a difference, effect or relationship to be true due to chance producing a particular state of events. Customarily the alpha level is set at 0.05, or, in no more than one in twenty statistical tests the test will show 'something' while in fact there is nothing. In the case of more than one statistical test the chance of finding at least one test statistically significant due to chance fluctuation, and to incorrectly declare a difference or relationship to be true, increases. In five tests the chance of finding at least one difference or relationship significant due to chance fluctuation equals 0.22, or one in five. In ten tests this chance increases to 0.40, which is about one in two. Thus we need to make an adjustment that will correct for multiple hypotheses. The Bonferroni method adjusts the alpha level of each individual test downwards to ensure that the overall risk for a number of tests remains 0.05. Even if more than one test is done the risk of finding a difference or effect incorrectly significant continues to be 0.05. To do this, it simply divides the alpha-level by the number hypotheses that were tested, so if 20 hypotheses were tested, then instead of using an alpha-level of 0.05, an alpha level of 0.0025 would be used. Alternatively, the p-values can be adjusted, by multiplying by the number of hypotheses that were tested, and the alpha-level can be kept the same. This approach is the one that GO::TermFinder takes. In the case of GO::TermFinder, the value used for the Bonferroni correction is the number of nodes to which the genes of interest are collectively annotated, excluding those nodes which only have a single annotation in the background distribution, which *a priori* cannot be significantly enriched. The Bonferroni correction assumes however that all hypotheses are independent. In the case of the GO::TermFinder, each hypothesis is a node in the Gene Ontology, which has two or more annotations (either directly or indirectly) from the tested group of genes (nodes with only one annotation are not tested). Because these hypotheses form a Directed Acyclic Graph (which is a subgraph of the full GO DAG) there are thus relationships between the hypotheses, meaning that they are not independent, and thus the Bonferroni correction may not be appropriate.

GO::TermFinder also includes a mode for correcting multiple hypotheses by running 1000 simulations. The corrected p-value is calculated as the fraction of simulations having any p-value as good or better than the observed p-value. Comparison of simulation corrected p-values with Bonferroni corrected p-values actually suggests that the Bonferroni correction is not conservative enough.

GO::TermFinder also calculate a False Discovery Rate, as a mean of sidestepping the issues of p-values and multiple hypotheses. Classic Multiple hypothesis correction can be very conservative, as it tries to maintain the probability of getting *any* false positives at a particular alpha level. The False Discovery Rate instead allows a user to choose a cut off that has an acceptable level of false discovery. Below is a example data generated from GO::TermFinder that allows comparison of corrected p-values and False Discovery Rate.

**Table 1**. Comparison of Bonferroni corrected p-values, simulation corrected p-values, and False Discovery Rate for the 28 most significant GO nodes, for a group of genes that show sensitivity to 1M NaCl and 10μM nystatin (Giaever et al, 2002; SNF7 STP22 VPS28 SNF8 VPS36 VPS25 YGR122W RIM20 RIM21 RIM8 RIM101 DFG16 RIM9 YGL046W RIM13 YNR029). Note that the Bonferroni correction is up to 2.8 fold *less* conservative than the simulation method that controls the Family Wise Error Rate. N/A – Not applicable – cases where no p-values better than that node’s p-value were seen in simulations.

| GO TERM | Rank | FDR (%) | EXPECTED FALSE POSITIVES | Uncorrected p-value | Bonferroni Corrected p-value | Simulation Corrected P-value | Simulation / Bonferroni |
| --- | --- | --- | --- | --- | --- | --- | --- |
| invasive growth (sensu Saccharomyces) | 1 | 0 | 0 | 1.93E-09 | 1.35343E-07 | 0.0001 | N/A |
| negative regulation of transcription by carbon catabolites | 2 | 0 | 0 | 1.25E-08 | 8.73665E-07 | 0.0001 | N/A |
| negative regulation of transcription by glucose | 3 | 0 | 0 | 1.25E-08 | 8.73665E-07 | 0.0001 | N/A |
| regulation of transcription by carbon catabolites | 4 | 0 | 0 | 1.25E-08 | 8.73665E-07 | 0.0001 | N/A |
| regulation of transcription by glucose | 5 | 0 | 0 | 1.25E-08 | 8.73665E-07 | 0.0001 | N/A |
| protein-vacuolar targeting | 6 | 0 | 0 | 2.36E-07 | 1.65203E-05 | 0.0001 | N/A |
| growth pattern | 7 | 0 | 0 | 4.45E-07 | 3.1173E-05 | 0.0001 | N/A |
| filamentous growth | 8 | 0 | 0 | 4.45E-07 | 3.1173E-05 | 0.0001 | N/A |
| protein processing | 9 | 0 | 0 | 4.97E-07 | 3.47607E-05 | 0.0001 | N/A |
| growth | 10 | 0 | 0 | 5.16E-07 | 3.60903E-05 | 0.0001 | N/A |
| cell differentiation | 11 | 0 | 0 | 4.11E-05 | 0.002873719 | 0.0062 | 2.157483003 |
| sporulation | 12 | 0 | 0 | 4.11E-05 | 0.002873719 | 0.0062 | 2.157483003 |
| cellular morphogenesis | 13 | 0 | 0 | 4.35E-05 | 0.003046811 | 0.0065 | 2.133378348 |
| morphogenesis | 14 | 0 | 0 | 4.35E-05 | 0.003046811 | 0.0065 | 2.133378348 |
| development | 15 | 0 | 0 | 5.86E-05 | 0.004100297 | 0.0115 | 2.804675065 |
| negative regulation of transcription, DNA-dependent | 16 | 0.125 | 0.02 | 0.000137524 | 0.00962667 | 0.0237 | 2.461910562 |
| negative regulation of transcription | 17 | 0.117647059 | 0.02 | 0.000147219 | 0.010305306 | 0.0238 | 2.309489955 |
| protein targeting | 18 | 0.111111111 | 0.02 | 0.000159899 | 0.011192922 | 0.0293 | 2.617725775 |
| cellular physiological process | 19 | 0.105263158 | 0.02 | 0.000219941 | 0.015395868 | 0.0352 | 2.286327771 |
| intracellular protein transport | 20 | 0.1 | 0.02 | 0.000229267 | 0.016048715 | 0.0359 | 2.236939238 |
| protein transport | 21 | 0.095238095 | 0.02 | 0.000261726 | 0.018320821 | 0.0416 | 2.270640585 |
| cellular process | 22 | 0.090909091 | 0.02 | 0.00029995 | 0.020996525 | 0.0429 | 2.043195292 |
| intracellular transport | 23 | 0.086956522 | 0.02 | 0.000473888 | 0.033172181 | 0.0635 | 1.91425462 |
| sporulation (sensu Saccharomyces) | 24 | 0.083333333 | 0.02 | 0.000566921 | 0.039684473 | 0.074 | 1.86470914 |
| sporulation (sensu Fungi) | 25 | 0.32 | 0.08 | 0.000726671 | 0.050866941 | 0.0905 | 1.779151603 |
| cell growth and/or maintenance | 26 | 0.615384615 | 0.16 | 0.00088497 | 0.061947893 | 0.1057 | 1.706272723 |
| protein-membrane targeting | 27 | 1.62962963 | 0.44 | 0.001412593 | 0.098881536 | 0.1373 | 1.388530208 |
| meiosis | 28 | 5.785714286 | 1.62 | 0.002936341 | 0.205543845 | 0.5511 | 2.681179779 |

In addition to providing tools for determining whether GO terms are associated with a list of genes at a significant level, this set of modules also defines an API for accessing and manipulating GO information. Below is a figure with a rough outline of the API.

**Figure 1. Simplified UML diagram of the architecture of GO::TermFinder and associated modules**.

Public methods defined by the abstract base class, GO::OntologyProvider, which are implemented by concrete subclasses, such as the GO::OntologyProvider::OntologyParser class that we have written, return either a single GO::Node, or an array of GO::Node instances. A subset of the public interface to GO::Node is shown, illustrating the various methods that exist to query the attributes of a GO::Node, as well as to traverse the GO structure.

**Other Tools With Similar Features**

There are many other tools that provide similar functionality to GO::TermFinder, in that they try to determine the level of significance for annotations on a set of genes. Below is a compiled list. If these have features that you think should be incorporated into GO::TermFinder to make it more useful, please send me email at sherlock@genome.stanford.edu.

| Tool | URL |
| --- | --- |
| CLENCH | http://www.personal.psu.edu/faculty/n/h/nhs109/Clench/ |
| EASE | http://david.niaid.nih.gov/david/ease.htm |
| FatiGO | http://fatigo.bioinfo.cnio.es/ |
| FunAssociate | http://llama.med.harvard.edu/cgi/func/funcassociate |
| FunSpec | http://funspec.med.utoronto.ca/ |
| GeneMerge | http://www.oeb.harvard.edu/hartl/lab/publications/GeneMerge/GeneMerge.html |
| GoMiner | http://discover.nci.nih.gov/gominer/ |
| GOSurfer | http://biosun1.harvard.edu/complab/gosurfer/ |
| OntoExpress | http://vortex.cs.wayne.edu/projects.htm#Onto-Express |
| OntologyTraverser | http://franklin.imgen.bcm.tmc.edu/rho/services/index.jsp?page=OntologyTraverser |
